# Understanding the interplay between organisational injustice and the health and wellbeing of female police officers: a meta-ethnography

**DOI:** 10.1186/s12889-024-20152-1

**Published:** 2024-09-28

**Authors:** Mahnoz Illias, Kathleen Riach, Evangelia Demou

**Affiliations:** 1 MRC/CSO Social and Public Health Sciences Unit, School of Health and Wellbeing, University of Glasgow, Glasgow, UK; 2 Adam Smith Business School, University of Glasgow, Glasgow, UK

**Keywords:** Gender, Health and wellbeing, Injustice, Meta-ethnography, Occupational health, Police, Qualitative, Sexism, Stress, Female police officer

## Abstract

**Background:**

Female police officers are reported to encounter more bias, discriminatory practices, and inadequate support than their male counterparts and experience poorer health outcomes. This meta-ethnographic review looks beyond individual responsibilities to consider which aspects of policing impact the health and well-being of female police officers.

**Methods:**

Primary qualitative and mixed method studies published between 2000 and 2024 were included. ProQuest (all databases) and Ovid (Medline and Embase) were searched using terms related to health, wellbeing, females, police, and qualitative research. This was a cross-jurisdictional review, with no limit on country of study. In total, twenty-one papers met the inclusion criteria. A seven-phase inductive and interpretative meta-ethnographic technique was employed to synthesise, analyse, and interpret the data.

**Results:**

The data analysis revealed a distinct outcome that demonstrated a strong relationship and substantial impacts of organisational injustice on the health and well-being of female police officers. Our findings showed that organisational injustice, encompassing procedural, relational, distributive, and gendered injustice, significantly influences the health and well-being of female officers. Impacts on mental health were commonly discussed, followed by aspects influencing social health, workplace wellbeing, and physical health. Moreover, the effects of these four forms of organisational injustice and the associated cultural, systemic, and structural risk factors extend beyond the immediate health and wellbeing impacts on the individual female officer through impeding other aspects of their work life, such as career progression and work-life balance, that can further impact long-term health and well-being**.**

**Conclusion:**

This review highlights the importance of addressing organisational injustice and the cultural, systemic, and structural risk factors within policing to promote healthier and more inclusive workforces for female officers. Policymakers and practitioners should critically examine policies and practices that may appear gender neutral but disproportionately impact women, affecting the health and well-being of female police officers. By addressing these issues, transformative action can be taken to create safer, more supportive, and healthier working environments for female police officers.

**Supplementary Information:**

The online version contains supplementary material available at 10.1186/s12889-024-20152-1.

## Background

Policing is one of the most challenging and stressful occupations. Police officers are constantly confronted with potentially dangerous situations and deal with physically, psychologically, and emotionally intense experiences [1–3]. Moreover, deeply entrenched organisational structures in policing also contribute to stressful and anxiety-inducing working conditions, leading to increased vulnerability among police officers to adverse psychosocial outcomes, such as stress [1, 4], anxiety [2, 4], posttraumatic stress disorder (PTSD) [1, 5], depression [2, 5], burnout [3, 4], somatisation [3, 4], and work-family conflict [4, 6, 7]. Despite showing high levels of attachment to their jobs, an increasing number of police officers across jurisdictions (e.g. in England and Wales [8, 9], the United States [10]) are choosing to exit their profession through avenues such as early voluntary retirement, which has been associated with organisational and occupational risk factors in different ways, including excessive workload, job strain, lack of autonomy, issues with leadership, and social support [8]. The organisational structure within policing has been identified as a significant stress risk [11, 12]. For female police officers, gender-based discrimination at work and work-life balance issues pose additional challenges that also impact retention rates [13].

While the number of female police officers is steadily increasing, policing remains a male-dominated profession [14]. In the UK, women make up just under a third of the workforce in England, Wales, and Scotland [15, 16]. While there is still significant progress to be made, the UK stands out as having a relatively higher representation of female police officers compared to the US [17, 18]. Jurisdiction may put measures in place to improve female representation, as such the US for instance has initiated the 30 × 30 initiative, which aims to boost the proportion of women in police recruits to 30% by 2030 [17, 18].

The obstacles faced by female police officers in advancing towards gender equality vary worldwide and are influenced by diverse historical, cultural, political, and policing contexts [19]. Nevertheless, global progress towards gender equality in policing is a continuing challenge. Global-South countries face substantial challenges for female police officers during the integration phase, whereas Western countries pose barriers during organisational transformation, characterised by male-dominated structures and the presence of the glass ceiling for leadership positions [19]. At the same time, female police officers have reported experiencing disproportionally adverse health experiences arising from organisational context [3, 4, 7]. Evidence suggests that their exposure to higher levels of workplace harassment, discrimination, bias, and lack of support based on their gender and minority status is a contributing factor [6, 20]. Previous studies report that female officers suffer disproportionately from psychosocial problems, such as stress, depression, burnout, PTSD, and work-family conflict, compared to their male counterparts [3–5, 7, 21, 22]. These psychosocial issues can in turn make them more susceptible to developing adverse physical outcomes, such as cardiovascular diseases, dyslipidaemia, hypertension, obesity [23], and reproductive health problems, including issues and disruptions with fertility, menstruation, and menopause [24, 25].

Systematic reviews have focused on different aspects of police health and wellbeing. For instance, one review highlighted how police officers are at an elevated risk for cardiovascular diseases, primarily due to the high levels of stress encountered in their line of work [26]. Other reviews have examined occupational hazards, injuries and diseases [27], trauma exposure, stigma associated with mental health, organisational stressors and mental wellbeing [28], risk factors for mental health [29], posttraumatic stress disorder [30], depression [31], sleep quality [32], lifestyle and health [33]. Despite existing research documenting the challenges faced by women over the years in policing [34], to date, less emphasis has been given to the systematic analysis of women's qualitative experiences of health and wellbeing in the broader context of policing. Such work is vital as qualitative work enables us to explore the influence of intricate cultural, systemic, and structural factors. There is a paucity of research providing a gender-sensitive account of organisational justice in terms of how it harbours or hinders the health and wellbeing of female police officers. This gap is significant because gender plays a pivotal role in shaping perceptions, practices, and implementations of justice within organisations. [35].

This review sets out to provide a new conceptual framework based on previous qualitative research to improve our understanding of the health and wellbeing of female police officers. We aim to identify the significance of the interplay between individuals, groups, communities, and the physical, social, and structural aspects of policing environments in terms of their effects on health and wellbeing. To do this, we have integrated the social-ecological model of health [36–39] along with ideas related to workplace wellbeing [40], focusing on gender-specific issues in policing. This approach allows a more nuanced examination of the contextual factors that may impact the health of this workforce. We conducted a critical examination of how organisational and operational risk factors within policing impact the health and wellbeing of female officers, considering perspectives from both male and female counterparts. This approach allows us to uncover overlooked dimensions of how organisational issues are perceived and experienced by female officers. This review adds to the existing body of knowledge but also attempts to shift the paradigm to include a more nuanced understanding to inform more effective, gender-responsive policies and practices to support health and wellbeing of female police officers.

## Methodology

**Fig. 1:**
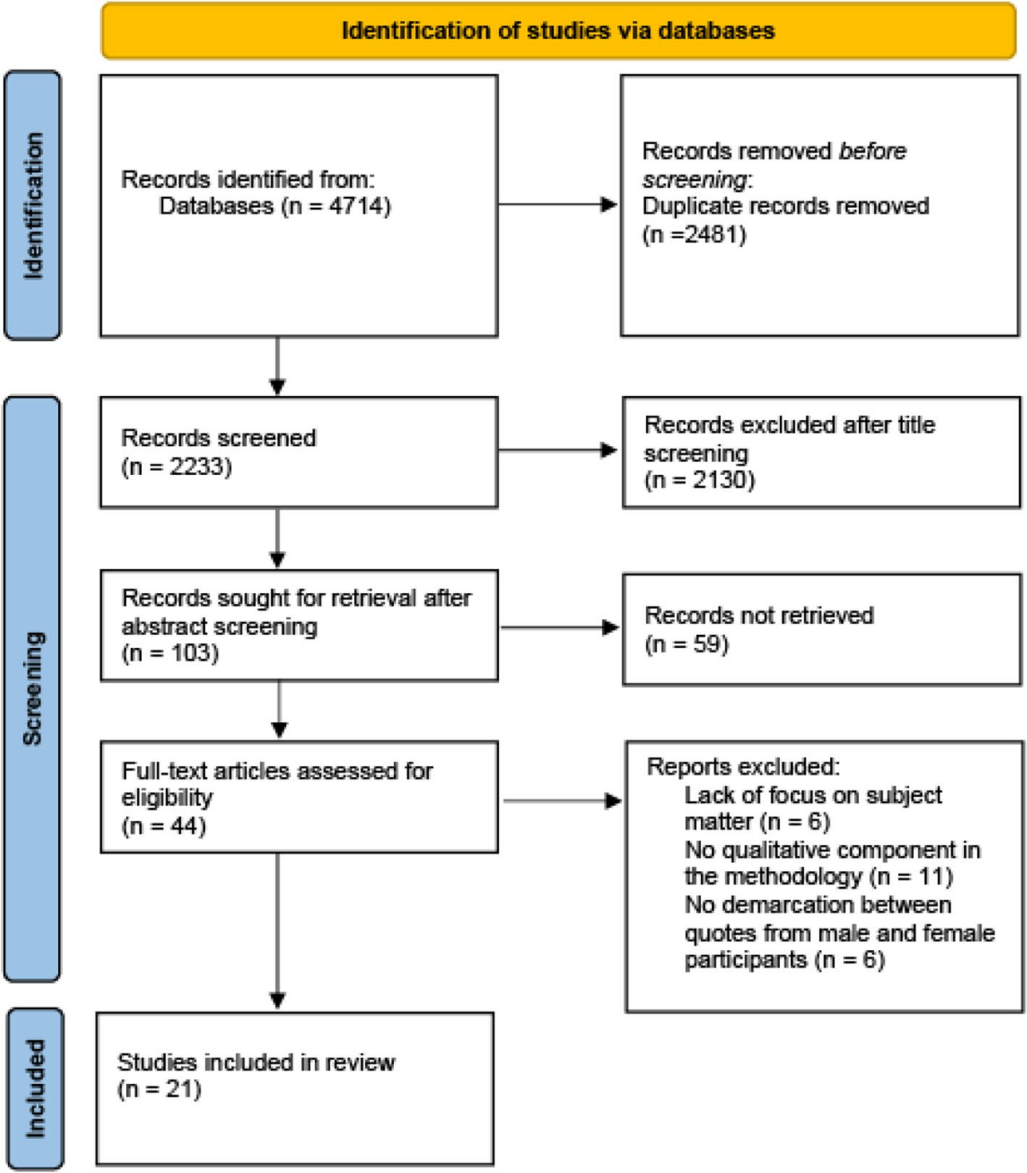
PRISMA Flow Diagram of Article Identification & Selection process

This study draws on existing international qualitative research published from 2000–2024 to understand the concepts and determinants of the health and wellbeing of female police officers. We have chosen this time-frame to ensure our findings are current but also to account for the many changes in policing during the last few years and how these have occurred at different times and rates across jurisdictions. The first author assessed the titles and abstracts of all records generated using the predefined inclusion and exclusion criteria and subsequently evaluated the full-text articles based on the same inclusion and exclusion criteria. (Fig. 1). A meta-ethnographic approach was undertaken due to its ability to synthesise qualitative studies focused on a specific phenomenon and its ability to mutually translate different studies into one another by transferring metaphors, ideas, and concepts across studies [41]. Meta-ethnography uses a seven-phase inductive and interpretative methodology that allows analysis to preserve the nature of relationships between themes within studies while creating new conceptual understandings rather than simply summarising existing studies [26, 41–43]. The study protocol was registered on the Open Science Framework (10.17605/OSF.IO/5F63H).

### Search strategy and identification of relevant studies

The original search was conducted by the first author in 2022 (26–02-2022) and was updated in 2024 (09–07-2024). Due to the interdisciplinary nature of research surrounding policing, ProQuest (all databases) and Ovid (Medline and Embase) were used to search for relevant articles. These platforms and databases were selected due to their relevance in covering articles related to health and policing. Search terms included synonyms, related terms, plural forms, and different spellings for the terms “Health & Wellbeing”, “Female”, “Police Officers” and “Qualitative Study” (see Figure S1).

### Inclusion/exclusion criteria

The reporting of the review followed PRISMA [27] and ENTREQ guidelines [44]. Systematic reviews, grey literature, and studies published in languages other than English were excluded from our review. At full-text screening phase, studies were excluded for not having qualitative components, insufficient focus on the subject matter (i.e., health and wellbeing of female police officers), or not focusing on women police officers’ experience, or studies that included both male and female officers but did not provide extractable information specific to females. We also omitted studies on the public’s perception or experiences with the police.

Eligible studies included peer-reviewed qualitative or mixed-method peer-reviewed articles in English published between 2000 and 2024. For mixed-method studies, only the qualitative components of the mixed-method studies were retrieved. The inclusion criteria encompassed studies involving exclusively female or mixed populations (both male and female). Articles containing perspectives from male police officers were only considered if they provided an insight regarding any health and wellbeing aspect impacting female police officers. It also included studies examining various aspects of physical, mental, and social health, and workplace wellbeing among female police officers.

### Quality appraisal

Following the identification of the corpus and application of inclusion and exclusion criteria, the first author assessed the full texts using the Critical Appraisal Skill Programme (CASP) [28] criteria for Qualitative Studies [29].

### Data synthesis and analysis

The eMERGe reporting guidance [42] was applied in this review to enhance reporting quality. NVivo 12 was used for data synthesis and analysis to ensure the systematic exploration of the data corpus. First, the lead author read the included articles multiple times. The other authors read a selection of papers each to ensure agreement of inclusion and of the analytical framework. Then, a customised standard template that was piloted and agreed upon by all co-authors was used to extract relevant study characteristics (Table 1). After discussion among the research team and before the analysis commenced, it was decided that all three approaches of synthesis, i.e., translational, refutational, and line of argument, would be combined to augment the outcomes of the review [42]. In the next phase of analysis, themes were extracted, translated, and integrated from individual studies and were systematically incorporated with themes from the other studies [42]. Reciprocal themes converged, and refutational themes diverged from each other and were organised into their conceptual categories and placed under new higher-level themes. These conceptual categories included first-order themes (i.e., themes emerging at the participant level) and second-order themes (i.e., themes emerging at the author level). This process was repeated continuously until all included studies were translated into each other. The first- and second-order concepts were documented by the lead author and were reviewed by the second and third authors. After discussions and analyses with the co-authors, the lead author performed additional synthesis to generate third-order themes (meta-themes) that offered new insights and interpretations derived from the translated concepts extracted from the first- and second-order themes. All themes were reviewed, discussed, and agreed upon among the co-authors.

**Table 1:** Study characteristics

Author(s) & Year	Population & Location	Data Collection	Setting	Method of Analysis	Aspects of Health and wellbeing investigated	Type of Injustice
Angehrn et al. (2021) [45]	17 Police Officers (9 Female and 8 Male Officers), Canada	Qualitative study: Semi-structured in-depth interviews	Police services across Saskatchewan	Thematic Analysis	Mental & Social health, Workplace wellbeing	Relational, Distributive, Gendered
Archbold & Schulz (2008) [32]	14 Sworn Female Police Officers, USA	Qualitative study: Face-to-face, structured interviews	A Midwestern municipal police agency	Thematic Analysis	Mental & Social health, Workplace wellbeing	Relational, Distributive, Gendered
Atkinson et al. (2020) [46]	1197 Female Police Officers in Peri & Post Menopause, UK	Mixed-method study: Online Survey (open & close ended questions)	3 Police Forces	Thematic Analysis	Physical, Mental, & Social health, Workplace wellbeing	Procedural, Relational, Distributive, Gendered
Bikos (2021) [47]	116 Police Officers (76 Female, 40 Male), Canada	Qualitative study: Semi-structured in-depth interviews	31 police services across Canada	Thematic Analysis	Mental & Social health	Relational, Gendered
Brown et al. (2019) [48]	9 Female Police Officers, USA	Qualitative study: Semi-structured in-depth face-to-face interviews	A large South-eastern police department	Thematic Analysis	Mental & Social health, Workplace wellbeing	Relational, Gendered
Bullock & Garland (2019) [49]	59 Police Officers (24 Female & 35 Male), UK	Qualitative study: Semi-structured in-depth interviews	Six constabularies in England (5) & Wales (1)	Thematic Analysis	Physical, Mental & Social health, Workplace wellbeing	Procedural, Distributive, Relational
Cordner & Cordner (2011) [50]	74 participants (42 Female Officers & 32 Male Police Chiefs), USA	Mixed-method study: Online Surveys	3 metropolitan counties in south-eastern Pennsylvania	Thematic Analysis	Physical & Social health, Workplace wellbeing	Procedural, Relational
Edwards & Kotera (2020) [51]	5 Police Officers (1 Female, 4 Male), UK	Qualitative study: Semi-structured in-depth interviews	Police Departments across the UK	Thematic Analysis	Physical, Mental & Social health, Workplace wellbeing	Procedural, Distributive, Gendered
Giwa et al. (2021) [52]	3 Female Police Officers, Canada	Qualitative study: Semi-structured in-depth interviews	Police agency in a medium-sized city	Intersectionality theory and inductive thematic analysis	Mental & Social health	Relational, Gendered
Gumani (2019) [53]	17 Police Officers (4 Female, 13 Male), South Africa	Qualitative study: Unstructured face-to-face & telephone interviews, diary entries	Police agencies across Vhembe district	Interpretative Phenomenological Analysis	Physical, Mental & Social health, Workplace wellbeing	Relational, Distributive
Kringen & Novich (2018) [33]	35 Police Officers (19 Female and 16 Male Officers), USA	Qualitative study: Semi-structured in-depth interviews	A metropolitan police department in the south‒west	Data-driven inductive Analytic coding	Mental & Social health	Procedural, Relational
Laverick et al. (2019) [54]	Over 250 Female Police Officers across UK,	Qualitative study: Focus Group Discussion	14 Force Areas across the UK	Thematic Analysis	Physical, Mental & Social health, Workplace wellbeing,	Procedural, Relational, Distributive, Gendered
Morabito & Shelley (2018) [55]	47 Female Police Officers, USA	Qualitative study: Semi-structured in-depth interviews	30 law enforcement agencies across 7 states	Grounded approach	Mental & Social health	Relational, Distributive
Murray (2020) [19, 56]	20 Police Officers (10 Male, 10 Female), Canada	Qualitative study: In depth open-ended interviews	A police agency in a populous province	Thematic Analysis	Physical & Mental health, Workplace wellbeing	Procedural, Relational, Distributive, Gendered
Newton & Huppatz (2020) [57]	18 mid-career Female Police Officers, Australia	Qualitative study: Semi-structured in-depth interviews	4 Australian police organisations	Thematic Analysis	Physical, Mental & Social health, Workplace wellbeing	Procedural, Gendered
Rabe-Hemp (2008) [30]	24 Female Police Officers, USA United States	Qualitative study: In depth open-ended interviews	Police Agencies from a Midwestern state	Grounded approach	Physical, Mental & Social health, Workplace wellbeing	Procedural, Relational, Gendered
Rabe-Hemp (2009) [31]	38 Female Police Officers, USA	Qualitative study: Structured Open-ended Interviews	Police Agencies Across USA	Grounded approach	Physical, Mental & Social health, Workplace wellbeing	Relational, Gendered
Todak et al. (2022) [58]	32 Female Police Officers, USA	Qualitative study: Semi-structured in-depth interviews	Elite Specialty Unit across the USA	Grounded approach	Mental & Social health	Relational, Gendered
Turner & Jenkins (2019) [59]	6 Police Officers (3 Female, 3 Male), USA	Qualitative study: Series of Semi-structured in-depth interviews	Police Departments across the UK	Interpretative Phenomenon logical Analysis	Physical, Mental & Social health, Workplace wellbeing	Gendered, Procedural
Yates et al. (2018) [60]	20 Female Police Managers, UK	Qualitative study: Semi-structured in-depth interviews	One police service based in a metropolitan area of the UK	Interpretative Phenomenon logical Analysis	Physical, Mental & Social health, Workplace wellbeing	Relational, Distributive, Gendered
Yu (2018) [34]	20 Female Police Officers, USA	Mixed-method study: Semi-structured interviews	10 federal law enforcement agencies	Thematic Analysis	Mental & Social health	Relational, Distributive, Procedural

## Results

### Description of papers

After applying the eligibility criteria, a total of 21 studies were included (Fig. 1). Most of the studies were only qualitative studies (*n* = 18) [30–33, 45, 47–49, 51–60], with a smaller number of mixed method studies (*n* = 3) [34, 46, 50]. The majority had a female only sample, although in 9 studies [33, 45, 47, 49–51, 53, 56, 59] the sample was both male and female. There was a predominance of studies from the United States (*n* = 9) [30–34, 48, 50, 55, 58], with the remaining from the United Kingdom (*n* = 6) [46, 49, 51, 54, 59, 60], Canada (*n* = 4) [45, 47, 52, 56] Australia (*n* = 1), and South Africa (*n* = 1) (Table 1). Using the CASP tool, the quality of all twenty-one articles was rated as high (Table S1, Additional File).

### Exploring health and wellbeing dimensions

Each of the papers reviewed in this study explored various dimensions of health, encompassing physical health, mental health, social health, and workplace wellbeing. These dimensions were examined as potential exposures, outcomes, or both (Table S2), highlighting their interconnectedness and multifaceted nature.

The included papers primarily focused on mental health, followed by social health, workplace wellbeing, and physical health. Mental health was defined here as a dynamic state of internal equilibrium which enables individuals to use their abilities in harmony with universal values of society, basic cognitive and social skills; ability to recognise, express and modulate one's own emotions, as well as empathise with others; flexibility and ability to cope with adverse life events and function in social roles; and harmonious relationship between body and mind represent important components of mental health which contribute, to varying degrees, to the state of internal equilibrium [61]. Studies discussed mental health both in relation to exposure to risk factors and as an outcome [48, 50, 51, 53, 54, 59, 60], or only as an exposure to risk factors [30, 33, 45, 47, 49, 52, 55–58], or only as an outcome [31]. The exposures varied, including gender-based discrimination, work-life balance, gendered culture, stigma, and lack of support, which impact mental health in policing. Common mental health outcomes encompassed stress, anxiety, depression, isolation, and feelings of being undervalued. Coping strategies, social support, and positive factors for success emerged as potential mitigating factors.

Here, social health was defined as a multidimensional concept comprising of social integration, social contribution, social coherence, social actualisation and social acceptance, enabling individuals to effectively engage within their community, actively contribute to it, maintain meaningful social connections, follow personal growth and experience acceptance by their social atmosphere [62]. Regarding social health, several studies examined it both as an outcome and exposure [34, 51, 57], whereas no papers exclusively investigated social health as the only primary outcome. Other studies discussed social health solely as an exposure [30–33, 45–51, 53–55, 58–60]. The included studies identified various exposures related to social health, including negative attitudes, discrimination, harassment, lack of role models, challenges with promotions and gendered division of labour. The identified exposures impacting social health were closely connected to the mental health outcomes mentioned earlier, emphasising the interconnectedness of these factors. However, limited research explicitly examined social health as an outcome.

Workplace wellbeing was defined here to encompass the entirety of the working experience, ranging from the physical environment's quality and safety to employees' perceptions of their work, working environment, work climate, and organisational structure [40]. A number of studies explored workplace wellbeing as an exposure but not as an outcome [30–32, 45, 46, 48–51, 53, 54, 56, 57, 59, 60]. Similar to mental and social health, workplace wellbeing in policing encompasses various exposures, including negative attitudes, discrimination, challenges in maintaining work-life balance, and feeling trapped in certain roles. These factors played a significant role in the development of mental health problems, dissatisfaction with one's career, and concerns regarding physical health among police officers.

Lastly, only one paper discussed physical health both as an exposure and an outcome [57]. Six studies examined physical health as an exposure [31, 32, 53, 58, 60, 61], and five studies assessed it as an outcome [47, 49, 50, 52, 56]. Exposures included experiences of traumatic incidences, the need to display physical force, overwhelming responsibilities, lack of physical preparation for fitness tests, challenges with childcare and work-life balance, difficulties with expressing breast milk, inadequate facilities, uncomfortable uniforms, and shift work. Outcomes included exacerbated physical symptoms of ailments by female officers who are going through menopause compared to female officers who are not going through menopause, and interconnectedness of physical and mental health such as stress manifesting in physical symptoms.

### Reporting of Meta-themes

Through translating and grouping the first-order and second-order themes across the 21 included papers, 22 subthemes emerged (Table 2, S2). While exploring the patterns across and between the subthemes, organisational injustice began to emerge as a key construct in the corpus that helped to connect a variety of subthemes describing the experiences and risk factors for women's health and wellbeing in policing. The data led to the introduction and use of a framework based on "organisational justice". “Organisational justice” was coined by French (1964) to describe individuals’ perceptions of fairness in organisations. In recent years, organisational justice has emerged as an important way to understand workers' health and wellbeing more generally [63–66], with evidence of a strong relationship between organisational injustice and adverse health and wellbeing effects [67].

**Table 2:** Summary of interpretations (with indicative first-order themes)

Third order themes (Meta-themes)	Second order themes	Quotes representing corresponding first-order themes
**Procedural Injustice**	**Obstacles to Recruitment, Selection & Retention of Female Police Officers:** Lack of tailored recruitment for female officers, policies acting as deterrent, dangerous work settings, lack of opportunities for advancement	*“I went to a women and policing symposium before I ever joined … Hearing [the female officers] speak was truly great but they did mention … [to] an audience full of women, that these are the requirements and the haircut [policy] was mentioned, and there were literally women who got up out of their seats and never came back.” -Female/male?* [33]
	**Existing Bureaucratic Processes & Policies:** Policy ignoring cultural sentiment, compliance considered equivalent to commitment for policing, supporting organisational values devaluing particular forms of gender, differences in views on policies targeting gender inequalities, no action taken after formal complaining	*“I’ll get promoted on my merits, but it will be perceived that I’ll get promoted because I’m a woman…I need my voice at the table.”(on introduction of quota for women) -*Female [57]
	**Structural Stigma:** Variations in policy implementation, lack of flexible working arrangements, seniority-based promotion, lack of practical support system, training and support for promoted posts, lack of knowledge and awareness on mental health issues, reluctance to attend nonmandatory trainings,inadequacy of existing framework to identify perfect managerial candidates	*“We have got a million different policies in place, but there’s always that fine print at the end of every policy that says, at the commander’s discretion. Whatever that policy says, the commander can overrule it because it doesn’t operationally suit his command. That is his card... get out of jail free. Absolutely, regardless of how logical it is, how supported it is, how much evidence base you can present for requiring that flexibility, if the boss doesn’t want to be flexible, he can just say, “it doesn’t operationally suit us.” -*Female [57]
**Relational Injustice**	**Shortcomings of Leadership & Management:** Window dressing, deshouldering the responsibility, imposing presenteeism, comparatively stricter scrutiny with female cadets, unwelcoming response from the supervisors, pressuring to switch from part-time to full time jobs, insight into leadership	*“I’d get phone calls to say “When do you think you’ll be back in?”, and “You know how short we are on the front line” and I felt bad that I was at home while my other colleagues were struggling, and they were probably getting injured as well, so…it was a lot to do with how I felt as well in terms of obviously being an extra body, or police officer, made a massive difference that time. -*Female [49]
	**Practices of Hegemonic Masculinity:** Female officers treated as a number, considered as incompetent, infantilisation, labelled and devalued for being ambitious, preference for masculine traits in female police officers, adopting macho attitude, Pressure to live up to the social construct, masculine-coded police work, not ‘real policing’, differences in acknowledging sexism by male officers	*“I just spoke to this on International Women’s Day. We had a panel and I think they wanted us to say everything is great and everything is fine, but it’s not. I still deal with leadership who will say, well, you can’t do that by yourself; I’ll send a guy with you. The same man at work would never get that treatment; he’d be able to go and do it himself. Simple small things, [such as] doing an alarm—that’s a call we do 10 times a day.”—*Female [52]
	**Sexual Teasing or Harassment:** Victim of sexual harassment, considering sexual harassment as a reciprocating behaviour, avoidance to report	*“I think all of our women are a community within themselves because there’s often discussions in the locker room about like, “I can’t believe that just happened,” or “I can’t believe that person said that to me,” and they’ll say, “That happened to me too.” -*Female [45]
	**Internalised Sexism:** Considering agency in gender expression insignificant, scrutinising undesirable characteristics in other female officers, Segregation from the feminine qualities, showing superiority towards other female officers, trying to hide negative emotion or information	*“I try to keep a very professional look, and that’s part of the reason I keep my hair pulled back in this bun because I have got long hair, and I don’t like some of these female officers that have all the foo foo hairdos. I haven’t witnessed it, but I would imagine they get treated more like or looked at as a woman first than an officer.” -*Female [31]
	**Female Officers doing Gender:** Being feminine & competent officer, dilemma of dualistic gender characteristics, brings balance to the job, empathetic, non-impulsive	*“I’m more feminine I would say. I mean I paint my fingernails purple. I’m not manly … I guess more on the feminine side, but I can hold my own.” -*Female [33]
	**Efforts of Assimilation:** Impersonating male colleagues, role entrapment, avoiding competition, overachievement strategy, using hierarchical structure, avoid breastfeeding at work despite facilities, not reporting to avoid working in an uncomfortable environment, difference in work environment in absence of macho figure	*“I also feel singled out when it comes time for promotion. Supervisors constantly tell me that I should participate in promotion. I don’t want attention drawn to the fact that I am a female cop—people are already aware of that. More attention makes it harder on me.” -*Female [32],
	**Navigating the Promotional Process:** Social support, influence of egalitarian female leader, taking legal action	*“Sometimes I think you have to talk about if something truly happens here that truly stresses you out, you need to talk to somebody. In addition, it is nice to have a husband who is a police officer officer because he understands exactly what is going on.” -*Female [30]
	**Navigating Isolation:** Isolation of female officers, not included in informal socialising outside work, accepting the isolation, building alternative networks	*“You know I have never been invited to go play golf […] I’ve never been invited on the fishing trips, and I have never been invited to the ballgame.”* -Female [30]
	**Navigating Acceptance from Team Members:** Acceptance influenced by external influence, accepted on past performance, sexual preference plays role into acceptance, difficulty to earn acceptance in particular department, surpassing the discrimination during crisis period	*“We were truly busy last year from a call out perspective. We had three shootings, three officer-involved shootings with my team. I had an officer that was shot last October. Therefore, we hit the ground running and it didn’t take any time at all for these guys to feel like ‘yup, she’s got it. She knows what she is doing.’ Therefore, internally I haven’t had any problems.” [SWAT Commander] -*Female [58]
	**Perceived Camaraderie:** Gender-neutral feeling, for long-term colleagues, dissatisfaction with the performance of the colleagues, backbiting the colleagues	*“I see most of the ladies that have been here a while band together. We know what it is like being one of a few women, so it is important to have others for support.” -*Female [32]
**Distributive Injustice**	**Institutional Backlash Against Female Police Officers:** Deprivation from organisational resources, treated as a 'Token Female Officer', crediting gender for career advancement, need to prove their worth, loss of morale & dedication, avoid asking for help in fear of getting judged, feeling of being sabotaged, negative impact on career	*“A male’s idea or opinion could come across as more important that a female. So when a male speaks about something, they’re listened to. In addition, a lot ofttimes when a female speaks [...] it’s overlooked or it’s like: “yeah, okay it’s a good idea but we’re not doing that”.* -Female [45]
	**Organisational & Operational Repercussions:** Feeling unbefitting after return to work following injury, experience of dys-appearance leading to self-stigma, underreported mental illness, negative impact on mind,	*“This affected me a lot because it was my first case … a case of a 7-year-old … one of the cases that I worked on when I started working. I had not yet worked on other cases to have knowledge of other causes of the rape of young children, and why they were affecting me in that manner.” -*Female [53]
	**Response to Evolving Life Situations:** Compromising career advancement for family, difficulties due to caregiving responsibilities, restrictions on family planning, nonfamily-friendly working policies	*“Well, most women with five-to-seven years of service are having children so they go ‘there’s no way I could do that job, the overtime is ridiculous and you travel.’ So it doesn’t work for the most part. When you get up to my level and you look around the room, the women in the room mostly don’t have children, are lesbians with no children, are single for whatever reason, or they had their children very, very young before they joined.” -*Female [56]
	**Existing Support Initiatives & their Impacts:** Variance in availability & utilisation of available support system, results of training on mental, initiatives to support LGBTQ community	*“Likewise, another officer commented he had seen a noticeable difference in the way line management’s attitudes were also changing. …the police are trying to do more, I think they are trying to recognise signs of stress in people, and what have you, that’s what they’re training line managers to do anyway, they are putting us on courses about stress management and things like that.”-*Female [51]
**Gendered Injustice**	**External perceptions of female police officers:** Bad ass woman on the front page, motivational figures, pressure to perform, gender perception by public, receiving negative attention, sexualisation of female officers	*“We would go to do our little manoeuvres in front of the parade. You could hear these gasps of mostly women saying, ‘it’s a girl!’” Another said “to citizens, it was more like seeing a white rhino at the zoo. Look honey, that’s a female motor officer.”-*Female [58]
	**Experiences related to reproductive events:** Impact on quality of life and work experience, stigma towards reproductive events, discussion on menopause considered hyperbolic, embarrassed to disclose in male-dominated spaces, perceived gendered ageism, taking detrimental decisions in fear of being stigmatised	*“‘Don’t be like some of these female officers and just get pregnant as soon as you go 10–8.’ Which means don’t get pregnant as soon as you first get on the road [patrol] and have to be at the front desk for 9 months. I looked at my one girlfriend, and we were like “Is this guy for fucking real?”—*Female [48]
	**Stigma Towards Mental Illness:** Developing self-stigma, increased risk from toxic work environment, normalising mental illness symptoms as ‘part of the job’, ostracisation, fear of being stigmatised, fear of breach of confidentiality	*“Although the organisation had an ‘official line’ on mental illness, the reality was quite different and that once a diagnosis of mental illness ‘got out [your] card was marked” -*Female [59]
	**Normalising sexism:** Making inappropriate jokes, normalised as a mean to develop camaraderie, accepted as embedded characteristics, sexism towards women leaders from male subordinates	*“When I first got hired, I had one of my sergeants tell me [...] he said: “Just so you know, I’m a sexist, like don’t be offended by it, but I’m a sexist””.* Female [45]

This finding led to the development of our four overarching meta-themes (third order themes) (Table 2 & S2) that deeply resonated with the conceptual aspects of injustice and played a central role in understanding the health and wellbeing of female police officers as explained in the included studies.

### Meta-theme 1-Procedural injustice

The first emerging theme was "procedural injustice". Procedural injustice refers to health and wellbeing as significantly influenced by formal procedures within an organisation that in principle are intended to ensure a fair and consistent decision-making process [68]. Key to this is the idea of transparency and how people experience a system or process as implemented [69].

The studies that explored dimensions of procedural injustice emphasised different aspects related to health and wellbeing, including social, physical, and mental health and workplace wellbeing [30, 33, 34, 46, 49–51, 54, 56, 57, 59]. Social health challenges included lack of organisational support, lack of family friendly policies, exclusionary policies followed closely by discussions on mental health related issues such as stress, anxiety, depression, job dissatisfaction, stigma towards mental illness, pressure to conform leading to self-doubt and sense of being constrained, stigmatisation.

Procedures and policies could also negatively influence women officers by not considering their gendered positioning or reproductive identities in terms of their health and wellbeing needs [33, 49]. This lack of consideration for gender-specific needs was reported to affect both their mental and social health in the workplace. The lack of gender-specific policies, such as targeted recruitment necessitated the consideration of alternative approaches, such as overlaying existing structures with initiatives specifically targeted towards female police officers or possible positive actions. However, it is important to note that this overlaying approach may have unintended consequences, including concerns expressed by female police officers that it leads to perceptions of credit given based on gender rather than competency [57]. This perception, in turn, could negatively impact their mental health and workplace wellbeing. While there was an emphasis on unbiased procedures surrounding, for example, recruitment and selection, it was suggested that current processes compromised the mental and social health of female officers due to existing structures based on traditional masculine normative trajectories that failed to account for the socio-biographical trajectories of female officers [50]. Other examples included the recognition that procedural fairness surrounding impartiality could disproportionately impact different groups, affecting their mental and social health. For example, policies regarding cutting hair short during training or induction periods were a deterrent for females joining policing and could impact recruitment or retention and affect their mental health through feeling as if they do not belong [33].

Lack of authority over operational decision-making processes involved elements of procedural injustice and was reported to disproportionately impacted female police officers’ health and wellbeing, compromising their workplace safety. For instance, police officers from small or rural agencies needed to respond to calls for service or patrol alone, which may be viewed as riskier for females than for males [50]. The original article describes how solo patrols could be 'intimidating' for women officers and our analysis interprets such situations as therefore likely to impact their mental and physical health, due to higher levels of stress and potential dangers while working alone. Furthermore, opportunities for advancement within police departments that required geographic mobility were considered to disproportionately hinder women who, due to a greater likelihood of caregiving responsibilities were less able to relocate [30]. This could leave them feeling limited in their career progression and face difficulties balancing work and family responsibilities, with subsequent impacts on their mental and social health and workplace wellbeing. While male officers felt police forces were taking adequate initiatives to bridge the gender inequality gaps in these aspects, female police officers felt differently [56].

Even when procedures were gender sensitive or recognised as gender neutral, unnecessary bureaucratic processes made access to available support difficult [53]. Structural stigma was identified as generating bias within procedural systems that could exacerbate or create negative health and wellbeing impacts on female officers. These included a lack of practical support systems [49, 53, 54], lack of training and support for promoted posts [54], lack of flexible working arrangements [30, 54, 57], and few debriefing sessions after traumatic incidents [53]. Furthermore, there was a perceived lack of knowledge and awareness about mental health within policing, further contributing to the overall detrimental effects on officers' wellbeing [47, 49, 51, 54]. Training modules on welfare or wellbeing issues for officers that were orientated towards women or were perceived as speaking to experiences more likely to impact them, such as training on flexible working arrangements or support experiences that could impact health and wellbeing (e.g., menopause, eldercare), were reported to be classified as nonmandatory, allowing line managers to opt-out [54].

### Meta-theme 2- relational injustice:

The concept of relational injustice refers to the extent to which supervisors are viewed as not treating their employees fairly [67, 70]. Studies that addressed elements of relational injustice consistently highlighted impacts on social health, including a lack of female role models, a gendered culture, and a non-inclusive environment [30–34, 45–50, 52–56, 58, 60]. The included articles in this review also explored the implications of relational injustice for mental health, workplace wellbeing and physical health. Negative male attitudes, sexual discrimination and harassment, lack of work-life balance, job frustration, fear of repercussions, stigma, and self-stigma all were reported to impact on mental health. Relational injustice underpinned coping strategies such as relocation, acting masculine, and seeking support, as well as reproducing a gendered division of labour, difficulties with childcare, role entrapment, hostile work environment, lack of support for disclosed mental health issues, and limited access to support services and practical assistance after injury. Furthermore, the impact on physical health emerged through unmanageable workload and the cultural stigmatisation of mental illness.

Relational injustice emerged in negative interactions with supervisors/line managers and peers, and included such as unfair treatment, discrimination, and harassment, which led to stress, anxiety, and isolation [47, 49, 51, 53, 57]. Supervisors and the management team were reported to fail as liaisons between officers and senior leadership or management (See Table 2) [49, 51, 53]. The impacts on physical and mental health were described through processes of imposed presenteeism and return to work without consideration of their conditions while they were on sick leave [47, 49]. It was reported that supervisors failed to provide support to female officers seeking part-time and flexible working options due to caregiving responsibilities [54, 57], despite formal policies in place, as final approval and implementation were dependent on the local ‘commander’s discretion’ [57]. These experiences impacted female officers' wellbeing, morale, goodwill, and commitment to policing [47, 49, 51]. Furthermore, women reported trying to assimilate themselves into masculinist policing culture and ways of relating to peers and supervisors that caused additional strain (See Table 2) [30–33, 56, 57, 60].

Navigating peer acceptance was reported as central to health and wellbeing but was described to be a complex process in policing [32, 48, 52, 58]. Acceptance from team members for female police officers was noted to depend on certain factors, such as taking leadership initiatives around diversity and inclusion, past performance, or even sexual preference (See Table 2). Female police officers also noted sexualised behaviour and harassment by colleagues involving physical contact and coercion (See Table 2) [48, 71]. These elements of relational injustice not only generated stress and anxiety but were also connected to a broader hostile environment that discussed disclosing any episodes that impacted health and wellbeing, particularly those of a gendered nature [32, 45].

Male officers' perceptions surrounding the existence of sexism in policing were an important factor and appeared to vary depending on their personal experience of having a relative working as a female police officer [56]. The lack of cultural fit into the highly masculinised environment reproduced masculine coding of attributes of an ideal police officer. The consideration of anything opposite as ‘feminine’ conflated with ‘weak’, and ‘incompetent’. This could be internalised by women leading to perceiving themselves as less competent than their male counterparts in terms of belonging and dignity [45, 46, 51, 60]. Female police officers also reported dilemmas in navigating rigid gender norms associated with both masculinity and femininity, struggling to relate comfortably to these traditional gender roles [48, 52]. A sense of vulnerability also led female officers adopting various coping mechanisms (See Table 2) [30, 31, 33]. However, there was no evidence of how effective this was as a strategy for offsetting harm to health and wellbeing and was reported to still lead to psychological distress, emotional exhaustion, and decreased job satisfaction [49, 51, 63], along with disruptions in social support networks, feelings of isolation [30–32], and impacts on career progression due to a lack of informal networking opportunities [56]. While some female police officers felt they had to accept this isolation, others utilised the increasing presence and strength of the female population within their workforces as a means to form alternative social networks, leaning predominantly on female officers for social support [56]. Female police officers also relied on continuous support from their social support systems (e.g., family members, supportive colleagues, supportive supervisors—both male and female) to compensate for the overall lack of support within policing [30, 34, 55]. Additionally, gaining more educational qualifications, seizing ‘Kairotic moments' (i.e. moments when marginalised populations can change or reproduce social, institutional, and discursive practices), guidance from mentors, and standing up for their rights were considered to help female police officers succeed in their careers [55].

### Meta-theme 3- distributive injustice:

The meta-theme of distributive injustice emerged in relation to how female police officers' health and wellbeing were connected to decision outcomes and resource allocations. The outcomes or resources distributed can be tangible (e.g. pay) or intangible (e.g. praise) [72]. When examining distributive injustice, several studies demonstrated an equal emphasis on discussions pertaining to social health, such as lack of female role models, gendered division of labour, difficulties with childcare, limited social support as well as mental health including negative attitudes, sexual discrimination and harassment, work-life imbalance, coping strategies (e.g. relocation, seeking support), the impact of gender-based harassment, discrimination, frustrations in the workplace and to a lesser extent highlighted associations of workplace wellbeing [32, 34, 45, 46, 49, 51, 53–56, 60]. Practices that created distributive injustice were discussed in terms of resistance to change in police culture, limited access to support services, unmanageable workload (See Table 2).

In some instances, distributive injustice indirectly impacted health and wellbeing. This included institutional backlash against female officers, repercussions of organisational and operational events on health, unsupportive responses to evolving life situations, and shortcomings of existing support initiatives and their impacts [45, 47–49]. Female police officers reported being deprived of organisational resources because of their gender [32]. They reported feeling treated as a ‘token’ and on display and they had to keep continually prove their worth by working hard or being better in the job, which reflected a lack of appreciation [30, 32, 48, 55, 58]. Moreover, it was repeatedly reported how credit for their success in career progression was attributed to their gender [30, 32, 45, 48, 58] or that they faced their career being sabotaged because of their gender [32]. Female officers reported losing morale and their dedication to the profession due to the favourable promotion of male figures, which further impacted their overall job satisfaction and mental health [48, 60]. This all led to feelings that increased efforts were needed just to maintain the baseline credibility that male officers were automatically afforded [30, 45, 48, 58].

The lack of resources allocated to particular events in their line of work were also found to have health and wellbeing repercussions. Upon return to work following injuries due to operational events, female officers reported not being well supported by their organisation, leading to feelings of exclusion [49]. This lack of support, in our interpretation, could exacerbate feelings of not belonging among female officers, as previously reported in two others of the included studies for this analysis [45, 60]. Moreover, female officers would suppress bodily symptoms around illnesses or reproductive health, such as pain or stress, as acknowledging it made them feel weak or led to self-blaming given the lack of resources for supportive workplace initiatives [46, 60].

In other instances, there were more tangible examples of distributive injustice, such as pay or financial considerations, which were associated with poorer workplace wellbeing outcomes. The lack of work-life balance and family-friendly policies [50], in combination with financial penalties for adjusted working times, e.g., part-time work, impacted maternity or caring patterns [45]. Flexible working was deemed to be not well supported by the existing policies and structures, as acknowledged by both male and female police officers [45, 56]. Also, female officers felt their freedom to plan a family was negatively influenced by policies such as pension plans, which carried consequences and implications adding to immediate financial strains [45].

Distributive injustice was also directly connected with the lack and/or variability of provision for health and wellbeing support and systems. Some officers reported it as adequate [51], while other support systems were framed as tokenistic or gender blind rather than addressing the particular health and wellbeing interests and needs of female officers [49]. These discrepancies surrounding availability also extended to the resources and provision of line manager/supervisor mental health management training [51].

### Meta-theme 4- gendered injustice:

Gendered injustice was a distinct mode of injustice that appears particularly important in shaping the health and wellbeing experience of female police officers. While gender differentially impacted the aforementioned modes of injustice, there was also clear evidence that being a female officer as a gendered subject was itself central to negative health and wellbeing experiences in policing. We understand gendered injustice here as underpinned by naturalised truth claims about women's bodies and biologically driven capabilities and historically sanctioned/instituted modes of injustice that emerged from institutionalised policing structures and identities. This included the perpetuation of stigma towards female mental illness, external perceptions of female police officers, experiences related to reproductive health events, and normalising sexism.

Several of studies spoke to elements of gendered injustice and its impact on social, mental, and physical health, and workplace wellbeing [30–32, 45–48, 51, 52, 54, 56–60]. Negative male attitudes, sexual discrimination, and harassment were identified as exposures leading to adverse mental health outcomes [34]. Participants highlighted how mental illness [46] was described as a ‘weakness’, ‘endemic’ [59], and even a ‘dead end’ [60]. Although awareness of the existence of mental health issues and understanding of what it is to be mentally healthy was clear, the hiding and normalisation of the existence of mental illness in policing were also accepted as the status quo [49, 59, 60]. As a result, female officers faced a double burden of stigma surrounding mental illness and gender through cynical responses, less empathetic behaviour, and negative stereotyping [47, 49, 51]. This led to the development of self-stigma, impeding recovery, contributing to underreporting of mental illness and preventing help seeking behaviour, as the potential consequences and gendered costs of reporting were perceived to be too high [47, 49, 51, 60]. Female officers also reported being afraid that their mental illness would be problematised through reference to their gender, as historically expression of emotions was frowned upon in policing [31]. Furthermore, the lack of awareness of available organisational support was also disclosed as a reason for not seeking help [59].

Gendered injustice was also impacted physical health experiences. These were described as destabilising an already conditional acceptance of female officers, for example, how pregnancy, menstruation, and menopause were problematised [30, 45, 46, 48, 54, 55]. Policewomen faced additional obstacles during these times due to the masculine totems of their profession [48, 54]. These challenges were described as impacting their quality of life [46], made them suppress health and body-related needs, such as not taking breaks to express milk at work [57], and even led to more detrimental considerations, such as considering terminating pregnancies to avoid condemnation or thwarting their careers [48].

The cumulative impact of sexist and gendered structures on social health and workplace wellbeing was evident. Female officers discussed learning over time to become desensitised to psychosocial risks such as inappropriate jokes or sexual teasing [30, 32, 45, 48, 55] or tolerating harassment to maintain their careers [58]. This was even the case when promoted, with female policing leaders even reporting facing sexism from male subordinates [56]. External stakeholders also influenced this. Female officers suggested that the public perceives them as police officers rather than ‘female police officers’ [31, 33]. The public considered female police officers to be women with exceptional calibres and role models for society [58]. While this recognition made female police officers feel good about themselves, it also led to increased pressure to perform, and without supportive conditions, this could be overwhelming [32, 58]. Conversely, some incidences of negative sexualisation of female police officers by the public were also reported, which made them feel uncomfortable and reinforced their ‘token’ status, as their male counterparts never received such attention from the public [32, 58].

## Discussion

### Line of argument: the consequences of injustice on female police officers' health and wellbeing

This review examined the health and wellbeing of female police officers in terms of mental, social, and physical health and workplace wellbeing. It considered this through inductively identifying a novel framework that understood health and wellbeing as exacerbated by different modes of organisational injustice, namely, procedural, relational, distributive, and gendered injustice. The findings show that the consequences of different forms of organisational injustice adversely affect not only the daily lives of female police officers but also their career progression and long-term health and wellbeing. This represents a new and valuable pathway in situating health and wellbeing as a systemic challenge in policing. This is significant given that organisational injustices can also influence a worker's trust in their organisation, threaten their self-worth, violate moral principles, and activate unhealthy responses [73]. Previous research has also suggested that low perceived justice is associated with factors that increase susceptibility to illness, such as high serum lipid levels and negative emotions [74–76]. This suggests that considering health and wellbeing as a justice issue in policing rather than solely a cultural or individual phenomenon, is central to creating a better-equipped, healthier, and more inclusive police force.

The benefits of viewing wellbeing as a justice issue are multifaceted. Firstly, it acknowledges that structural and systemic factors play a critical role in the health outcomes of female police officers, thereby shifting the focus from individual resilience to organisational accountability. This perspective can lead to more comprehensive and effective policy interventions aimed at reducing systemic inequalities, as supported by research in broader contexts outside of policing [77]. Secondly, it promotes a more holistic understanding of wellbeing, thereby fostering a more supportive and inclusive workplace culture [77].

Working towards better health and wellbeing for female police officers requires tackling broader systems of relational injustice. The masculinist parameters of conduct and their negative implications for health and wellbeing reported in this review, resonate with the findings of previous research [78–81] reporting how organisational and operational stressors affect the psychological wellbeing of police officers more generally. However, our study emphasised how different modes of injustice have significant implications for female police officers' health and wellbeing. This is both in providing the conditions for experiences that adversely affected their health and wellbeing and foreclosing possibilities to challenge behaviours that led to suppression and creating stressful and hostile environments not conducive to positive health and wellbeing.

Moreover, the different forms of organisational injustice do not exist in isolation but often overlap and interact, compounding their effects on female officers. For example, gendered injustice often intersects with procedural and relational injustice. Female officers experiencing gender-based discrimination may also face procedural barriers when seeking promotions or fair evaluations, as well as relational difficulties such as lack of support from colleagues and supervisors. These intertwined injustices can create a compounded negative effect on overall wellbeing. Additionally, distributive injustice, such as unequal pay or lack of recognition, can exacerbate relational injustices, leading to strained relationships and reduced social support within the workplace. Procedural injustices, like unfair evaluation processes, can undermine trust in leadership and further entrench relational and gendered injustices. Understanding these overlaps is crucial for developing comprehensive interventions that address multiple dimensions of injustice simultaneously.

Female police officers may be ensconced by the hypermasculinised ideals and attitudes of policing; however, these same ideals and attitudes also restrict their role and limit their acceptance. While negotiating these relational forms of injustice may give some female police officers self-satisfaction, it also led to feeling pressured and stressed to live up to the expectations imposed by the historical social constructs of women in policing [45, 52]. Due to the existence of macho-cultures, the stigma around the expression of emotional responses to both mental and physical illness was evident. This significantly impacted the help-seeking behaviour of female police officers, as it was considered a sign of weakness or professional incompetence. Female police officers themselves maintained the hegemonic masculinity of policing culture by adopting role entrapment [82]. The wellbeing experiences of female police officers were greatly impacted by their traditional caregiving role in society and were compelled to move to job roles more accommodating to family life (e.g., part-time work, administrative jobs), which subsequently jeopardised their career prospects. The existence of relational injustice was also reflected through leadership attitudes, where female officers reported facing inadequate supervision, poor relationships with supervisors, and lack of support, which affected their mental wellbeing. However, despite these challenges, social support systems were deemed key in providing female officers with the support and opportunities to advance their careers.

Our findings give valence to previous studies of occupational health that have highlighted how procedural and relational injustice are associated with increased risk of mental illnesses, sickness absence, self-reported stress reaction, and poor perceived health status [63, 66, 70, 74]. In addition, it shows how perceived challenges in accessing organisational resources, advancement opportunities, tokenism, and cynical behaviour from colleagues and supervisors adversely impact female officers' health and wellbeing. This was due to not receiving timely and gender-sensitive support and being pulled into ways of working that were not as financially rewarding or have limited career advancement opportunities.

Our analysis demonstrated that health and wellbeing were also heavily influenced by an infrastructure that reproduced gendered modes of injustice that were commensurate with normative parameters of what it means to police. Female officers bore a burden of traditional views or normative ideas about female police officers that resulted in overwork to meet basic acceptance, their bodies viewed as problematic, always subject to being a 'female' police officer, and the conditional parameters of acceptance associated with this.

This review also highlighted that the reproductive health experiences of female officers were impacted by their occupation. In some cases, female officers were discriminated against based on their physiological changes during the female life course, including during pregnancy, menstruation, and menopause. However, the data on reproductive and physical health (e.g., operational injuries) were limited, and is an area that requires further exploration.

### Limitations

To the best of our knowledge, this is the first meta-ethnographic review on the health and wellbeing of female police officers that inductively developed a valuable lens to depart from individually focused explorations of gender and health and wellbeing in police work. However, there are invariably some limitations. Although our literature search was systematic, comprehensive, and up to date, the disparate nature of exploring elements of health and wellbeing studies means that some studies may have been missed due to search criteria. Though qualitative research excels in exploring complex, multifaceted phenomena, it sometimes lacks reporting of the detailed analytical processes involved. Although, using the CASP tool, all the articles were found to be of high quality. However, the CASP scores have also highlighted potential flaws, such as studies not reflecting on the potential bias of the researcher or not reporting aspects related to ethical considerations [47, 50, 54, 56]. For some studies, it was not possible to conclude whether the results would have a local impact [32–34, 46, 50–56]. Moreover, synthesising findings obtained through various qualitative methods across the included articles may be a limitation as previously reported [83, 84]. Another potential limitation of this review is the small sample size of certain included studies [45, 48, 51–53, 59], which may limit the generalisability of the findings beyond the specific contexts examined. The restricted number of participants in individual studies could constrain the range of perspectives represented, potentially impacting the depth and comprehensiveness of the data analysis. In the context of this review, it is noteworthy that nine of the articles included in the analysis encompassed a sample population comprising both male and female police officers. These studies allowed for the identification of first-order themes specifically pertaining to the health and wellbeing of female police officers, as well as insights derived from male police officers regarding the same topic. However, given that many experiences in policing can be shared by both genders, the second-order themes arising from these studies might be relevant to the experiences of both male and female police officers.

## Conclusion

This study represents a novel effort in examining the health and wellbeing of female police officers by conducting a meta-ethnographic systematic review of the qualitative literature. The findings shed light on the systemic challenges faced within the policing context and highlight the need for comprehensive actions to promote equitable and healthier workforces. The identified modes of injustice, including procedural, relational, distributive, and gendered injustice, in addition to exacerbating adverse health and wellbeing outcomes have significant implications for both job performance and long-term career trajectories of female police officers. To address these issues, multilevel interventions should be considered. Among the most critical actions is the implementation of transparent services and gender-neutral policies, as these can mitigate procedural and distributive injustices by ensuring fair treatment and equal opportunities for all officers. Providing support for reproductive health experiences is another crucial measure. This includes accommodating pregnancy, menstruation, and menopause through appropriate policies, which can alleviate physical and mental stress, thereby improving overall wellbeing and job satisfaction. Flexible working arrangements are vital for addressing work-life balance issues, particularly for female officers with caregiving responsibilities. These arrangements can help retain talent and reduce the pressure that contributes to job strain and burnout. Addressing gender-based violence within the police force is paramount. Establishing strict protocols and providing training on recognising and preventing such violence can create a safer and more supportive environment for female officers. Additionally, fostering a culture of social support through mentoring programs and peer support networks can help female officers navigate the unique challenges they face and reduce feelings of isolation and exclusion. Further large-scale, and longitudinal research is required to better understand the differential impact of social determinants on the physical, mental, and social wellbeing of female police officers.

## Supplementary Information

Supplementary Material 1.

## Data Availability

Please contact the corresponding author with requests.
