# Supplementary material for: Understanding the interplay between organisational injustice and the health and wellbeing of female police officers: a meta-ethnography
Source: BMC Public Health. 2024 Sep 28;24:2659. doi: 10.1186/s12889-024-20152-1 (PMC11439285; doi:10.1186/s12889-024-20152-1)
Supplement: Supplementary file 1 — Supplementary Material 1. [file 12889_2024_20152_MOESM1_ESM.docx]

**Understanding the Interplay between Organisational Injustice and Health and Wellbeing of Female Police Officers: A Meta-ethnography**

**[Supplementary Material]**

Mahnoz Illias^1*,^ Evangelia Demou^1^, Kathleen Riach^2^

1. MRC/CSO Social and Public Health Sciences Unit, School of Health and Wellbeing, University of Glasgow, Glasgow, UK

2. Adam Smith Business School, University of Glasgow, UK

*Corresponding author:

Mahnoz Illias

MRC/CSO Social and Public Health Sciences Unit,

School of Health and Wellbeing,

University of Glasgow,

Glasgow, UK

Email: [m.illias.1@research.gla.ac.uk](mailto:m.illias.1@research.gla.ac.uk)

**Table S1: Quality assessment of articles using CASP tools**

**Supplementary Materials- Quality assessment of articles using CASP tools**

| **Authors & Years** | **Section A: Are the results valid?** | | | | | | **Section B: What are the results?** | | | **Section C: Will the results help locally?** | **Total score** |
| --- | --- | --- | --- | --- | --- | --- | --- | --- | --- | --- | --- |
|  | Q1 | Q2 | Q3 | Q4 | Q5 | Q6 | Q7 | Q8 | Q9 | Q10 |  |
| Angehrn et al., 2021 | Yes | Yes | Yes | Yes | Yes | Yes | Yes | Yes | Yes | Yes | 20/20  High |
| Archbold & Schulz, 2008 | Yes | Yes | Yes | Yes | Yes | Can’t Tell | Yes | Yes | Yes | Can’t tell | 18/20  High |
| Atkinson et al., 2020 | Yes | Yes | Yes | Yes | Yes | Yes | Yes | Yes | Yes | Can’t tell | 19/20  High |
| Bikos, 2021 | Yes | Yes | Yes | Yes | Yes | Can’t tell | Can’t tell | Yes | Yes | Yes | 18/20  High |
| Brown et al., 2019 | Yes | Yes | Yes | Yes | Yes | Yes | Yes | Yes | Yes | Yes | 20/20  High |
| Bullock & Garland, 2019 | Yes | Yes | Yes | Yes | Yes | Yes | Yes | Yes | Yes | Yes | 20/20  High |
| Cordner & Cordner, 2011 | Yes | Yes | Yes | Yes | Yes | Can’t tell | Can’t tell | Yes | Yes | Can’t tell | 17/20  High |
| Edwards & Kotera, 2020 | Yes | Yes | Yes | Yes | Yes | Can’t Tell | Yes | Yes | Yes | Can’t tell | 18/20  High |
| Giwa et al., 2021 | Yes | Yes | Yes | Yes | Yes | Can’t tell | Yes | Yes | Yes | Can’t tell | 18/20  High |
| Gumani, 2019 | Yes | Yes | Yes | Yes | Yes | Yes | Yes | Yes | Yes | Can’t tell | 19/20  High |
| Kringen & Novich, 2018 | Yes | Yes | Yes | Yes | Yes | Yes | Yes | Yes | Yes | Can’t tell | 19/20  High |
| Laverick et al., 2019 | Yes | Yes | Yes | Yes | Yes | Can’t tell | Can’t tell | Yes | Yes | Can’t tell | 17/20  High |
| Morabito & Shelley, 2018 | Yes | Yes | Yes | Yes | Yes | Yes | Yes | Yes | Yes | Can’t tell | 19/20  High |
| Murray, 2021 | Yes | Yes | Yes | Yes | Yes | Yes | Can’t tell | Yes | Yes | Can’t tell | 18/20  High |
| Newton & Huppatz, 2020 | Yes | Yes | Yes | Yes | Yes | Yes | Yes | Yes | Yes | Yes | 20/20  High |
| Rabe-Hemp, 2008 | Yes | Yes | Yes | Yes | Yes | Yes | Yes | Yes | Yes | Yes | 20/20  High |
| Rabe-Hemp, 2009 | Yes | Yes | Yes | Yes | Yes | Yes | Yes | Yes | Yes | Yes | 20/20  High |
| Todak et al., 2022 | Yes | Yes | Yes | Yes | Yes | Yes | Yes | Yes | Yes | Yes | 20/20  High |
| Turner & Jenkins, 2019 | Yes | Yes | Yes | Yes | Yes | Yes | Yes | Yes | Yes | Yes | 20/20  High |
| Yates et al., 2018 | Yes | Yes | Yes | Yes | Yes | Yes | Yes | Yes | Yes | Yes | 20/20  High |
| Yu, 2018 | Yes | Yes | Yes | Yes | Yes | Yes | Yes | Yes | Yes | Can’t tell | 19/20  High |

The articles have been marked as “Low”, “Moderate” and “High” depending on the CASP Tool and the judgement of the first author.

Yes= 2, Can’t tell =1 and No= 0, Highest possible total score= 20

If total score, 15-20: High

If total score, 10-14: Moderate

If total score, 0-9: Low

Low- 0 articles

Moderate-0 articles

High- 21 articles

**Table S2. Full summary of interpretations**

| Third order themes | Second order themes | Quotes representing corresponding first order themes |
| --- | --- | --- |
| Procedural Injustice | **Obstacles to Recruitment, Selection & Retention of Female Police Officers:**  Lack of special recruitment attempts targeting female police officers,  Policies acting as deterrent,  Dangerous work settings,  Lack of opportunities for advancement. | “We advertise for police officers. Period. We don’t do anything special to attract any class of person—women, men, white, black, Hispanic, etc.”-Male, Cordner & Cordner, 2011.  “I went to a women and policing symposium before I ever joined … Hearing [the female ofﬁcers] speak was really great but they did mention … [to] an audience full of women, that these are the requirements and the haircut [policy] was mentioned, and there were literally women who got up out of their seats and never came back.” -Kringen & Novich, 2018.  “This is a very dangerous career.” -Male, Cordner & Cordenr, 2011. |
|  | **Existing Bureaucratic Processes & Policies:**  Double-edged sword,  Making work difficult,  Policy ignoring cultural sentiment,  Compliance considered equivalent to commitment for policing,  Supporting organisational values devaluing particular forms of gender,  Differences in views on policies targeting gender inequalities,  No action taken after formal complaining. | “I’ll get promoted on my merits, but it will be perceived that I’ll get promoted because I’m a woman...I need my voice at the table.”(on introduction of quota for women) -Female, Newton & Huppatz, 2020.  “I’m just, like, it’s just hair. It will grow back’, she stated. ‘I mean, if you want something bad enough, you’re gonna do what it takes to make the sacriﬁces to get what you want.” -Female, Kringen & Novich , 2018.  “[Agency] has made great strides in issues surrounding gender, gender inequality and the like.”-Female, Murray, 2021.  “we said okay we are considering having families. Could we be assigned to desk duty during our pregnancy and our job. And his answer was no, I would have to ﬁre you if you couldn’t go on and do your job.” - Female, Rabe-Hemp, 2007. |
|  | **Structural Stigma:**  Variations in implementation of the policies,  Lack of flexible working arrangements,  Seniority-based promotion,  Lack of Practical Support System,  Reluctance to attend non-mandatory trainings,  Lack of training & support in relation to promoted posts,  Lack of knowledge & awareness about mental health,  Inadequacy of existing framework to identify perfect managerial candidates. | “We have got a million different policies in place, but there’s always that fine print at the end of every policy that says, at the commander’s discretion. Whatever that policy says, the commander can overrule it because it doesn’t operationally suit his command. That is his card . . . get out of jail free. Absolutely, regardless of how logical it is, how supported it is, how much evidence base you can present for requiring that flexibility, if the boss doesn’t want to be flexible, he can just say, “it doesn’t operationally suit us.” -Female, Newton & Huppatz, 2020.  “Occupational Health didn’t offer me any support; they didn’t point me in any directions of anybody. They basically said they haven’t got the funding anymore to do anything”. - Female, Bullock & Garland, 2019.  “I will be going through the menopause shortly, and I don’t know what to expect. I don’t know what [name of Force] will do about it. I don’t know how they will support it. There are times in your service where you will go through children, and you don’t know what to expect… You go through it and within the police service you tend to stay for 30 years, it is now going to be 35 of course. You need to be investing in your employees and going along this life-cycle journey with them and managing men and women, because we all have different needs at different ages and over the cycle of 35 years, you would expect to be a little bit up and down when you peak performance and when you drop performance, but it is about the overall picture really.” - Female, Laverick et al., 2019. |
| Relational Injustice | **Shortcomings of Leadership & Management:**  Window Dressing,  Deshouldering the responsibility,  Imposing presenteeism,  Comparatively stricter scrutiny with female cadets,  Unwelcoming response from the supervisors,  Pressuring to switch from part-time to full time jobs,  Insight into leadership. | “We have all the programs that you could get. We have Road to Mental Readiness. I was part of the initiation of the Critical Incident Stress Management and Peer Support teams. Honestly, it’s all just window dressing. I had one member about to commit suicide at the end of last year. I bring it upstairs and say, “I don’t know what to do to help them.” The Chief didn’t like this officer, blamed them, and did nothing. So, I’ve seen it first-hand–we have everything we need but we don’t have buy in from the top and therefore, it’s not trickling down.”- Female, Bikos, 2021.  “I’d get phone calls to say “When do you think you’ll be back in?”, and “You know how short we are on the front line” and I felt bad that I was at home while my other colleagues were struggling, and they were probably getting injured as well, so…it was a lot to do with how I felt as well in terms of obviously being an extra body, or police oﬃcer, made a massive diﬀerence that time. -Female, Bullock & Garland, 2019.  “It’s the group of senior leaders. They think they know everything. In this case it is a gender thing. They tend to be men because there’s not a whole lot of women in that world. They tend to be forty-year-olds because that’s who fulfills those roles right now. We actually had women acting in those roles and they weren’t like that.” -Female, Murray, 2021.  “No different than having a man as a supervisor. If there’s any difference, it’s probably a positive rather than a negative. She’s a bit more accommodating than some men would be.”-Male, Murray, 2021. |
|  | **Practices of Hegemonic Masculinity:**  Female officers treated as a number,  Considered as incompetent,  Infantilisation,  Labelled and devalued for being ambitious,  Preference for masculine traits in female police officers,  Adopting macho attitude,  Pressure to live up to the social construct,  Masculine-coded police work,  Not ‘real policing’,  Differences in acknowledging sexism by male officers. | “When I was first hired there were many references made to the fact that I was one of only a few women in the department. More recently, they have hired more females. It is like they want women in the department so badly that they will hire as many as often as they can regardless of their qualifications. I feel like I am a statistic sometimes because the department focuses on how many women, not who the women are as people.” -Female, Archbold & Schulz, 2008.  “So, I mean in terms of like, the promotions . . . like there’s always someone who has to decide whether you get the job or not, right. Like even the promotion level, you’ve got like your test that you write which, that’s black and white, but then you’ve got your assessment from your sergeant, and you have your assessment from your inspector. So, all ofthose things, its all, you know it’s all biased in a sense, right? Whether they like you or don’t like you, or you know. [ . . ] It’s harder for women because there’s a ﬁne line between being assertive and then everyone thinking that you’re a bitch and that kinda thing. So, does that affect your future stuff? Probably.”-Female, Angehrn et al., 2021.  “I just spoke to this on International Women’s Day. We had a panel and I think they wanted us to say everything is great and everything is fine, but it’s not. I still deal with leadership who will say, well, you can’t do that by yourself; I’ll send a guy with you. The same man at work would never get that treatment; he’d be able to go and do it himself. Simple small things, [such as] doing an alarm—that’s a call we do 10 times a day.” - Female, Giwa et al., 2021. |
|  | **Sexual Teasing or Harassment:**  Victim of sexual harassment,  Considering sexual harassment as a reciprocating behaviour,  Avoidance to report. | “I think all of our women are a community within themselves because there’s often discussions in the locker room about like, “I can’t believe that just happened,” or “I can’t believe that person said that to me,” and they’ll say, “That happened to me too.” -Female, Angerhn et al., 2022.  “Harassment is when one party isn’t enjoying the teasing, that person is being speciﬁcally targeted to make them feel uncomfortable or make them feel like they are less than something. Teasing is both parties are mutually involved in it.” - Female, Brown et al., 2019.  “But then it was like, okay, just ignore them, ignore them, ignore them, ignore them. And then it’s like text messaging, comments. And it’s, I don’t know, covert? It’s like, you know how it starts out, right? And then it would get to like some inappropriate comments, like even of superiors as you get into the organization. [ . . . ] And it was for me shocking and inappropriate. And again, we police but yet, allow that behavior?”-7, Female, Angerhn et al., 2022. |
|  | **Internalised Sexism:**  Considering Agency in Gender Expression Insignificant, Need to Create Equal Footing between Male & Female Cadets, Scrutinising Undesirable Characteristics in Other Female Officers, Segregation from the Feminine Qualities, Showing Superiority towards other Female Officers, trying to hide negative emotion or information. | “I try to keep a very professional look, and that’s part of the reason I keep my hair pulled back in this bun because I have got long hair and I don’t like some of these female officers that have all the foo foo hairdos. I haven’t witnessed it, but I would imagine they get treated more like or looked at as a woman first than an officer.” -Female, Rabe-Hemp, 2009.  ‘[My boyfriend] made fun of me, he would tell me I was a cute little boy. When he’d make fun of me, I’d be like just shut up, whatever, leave me alone, it’ll grow back.’ -Female, Kringen & Novich, 2018. |
|  | **Female Officers doing Gender:**  Being feminine & competent officer,  Dilemma of dualistic gender characteristics,  Brings balance to the job,  Empathetic,  Non-impulsive. | “I’m more feminine I would say. I mean I paint my ﬁngernails purple. I’m not manly … I guess more on the feminine side, but I can hold my own.” -1, Female, Kringen & Novich, 2018.  “You know men and women police differently. I think men will make a lot of decisions very, very quickly and that processing time sometime has to happen. Even though in a high stress situation sometimes you don’t have that time. I found women process things a little bit differently, especially when they’re newer to the job. They process a little bit differently. They make less snap decisions. They’re not as willing to go to a physical sort of confrontation, which is good. Generally, I ﬁnd them easier to work with. I work way better with women than I do with men. And I like the balance that we have.” -Male, Angehrn et al., 2021.  “A police officer said to me ‘you embody what a police officers should be,’ I go out of my way to help other people, I’m an effective communicator, people have reported my writing to be very good.” - Female, Murray, 2021. |
|  | **Efforts of Assimilation:**  Impersonating male colleagues,  Role entrapment,  Avoiding competition,  Overachievement strategy,  Using hierarchical structure,  Avoid breastfeeding at work despite having breastfeeding room,  Not reporting to avoid working in an uncomfortable environment,  Difference in work environment in absence of Macho figure, | “[Historically], certainly the male ofﬁcers thought that women were just there to look after the babies, to go and deal with the rape victims, to make the tea and do things like that … It’s not like that now, but it was very like that, and you had to prove yourself on the shifts in those days. And until you’d done that, they wouldn’t accept you.”-Female, Yates et al., 2018.  “I also feel singled out when it comes time for promotion. Supervisors constantly tell me that I should participate in promotion. I don’t want attention drawn to the fact that I am a female cop—people are already aware of that. More attention makes it harder on me.” -Female, Archbold & Schulz, 2008.  “You never let anybody call you by your first name if they’re a lower rank than you on the road. Over beer, who cares, but workwise you are the boss. 99 percent of the time it’s not necessary, but 1 percent [of people] need to follow the rules so they know to obey when you give directions.” -Female, Murray, 2021.  “I do not have a commanding male presence when I walk in. So I approach things differently.” -Female, Rabe-Hemp, 2009. |
|  | **Navigating the Promotional Process:**  Social support,  Influence of egalitarian female leader,  Taking legal action. | “Sometimes I think you have to talk about if something really happens here that really stresses you out, you need to talk to somebody. And it is nice to have a husband who is a police ofﬁcer because he understands exactly what is going on.” -Female, Rabe-Hemp,2007.  “We won $650,000 for the lawsuit, which was the largest pay out for a sex discrimination case at that time….[the chief] had no choice in the matter, he was ordered by the mayor at that point [to stop discussing anyone’s weight]. But he, you know, he was always right there in the background the whole time, and he still is, he’s still there. But, I mean, I grew around that and . . . I became the secretary on the union, and I did it mainly because I sat right across from him every single meeting, and I just sat there and stared at him at every meeting. And it was kind of like my way of making him uncomfortable…I had come to terms with the fact that I was just going to blow my career up, is what I thought. . . . I didn’t think anything good was going to come of it.”-Female, Morabito & Shelley, 2018. |
|  | **Navigating Isolation:**  Isolation of female officers,  Not included in informal socialising outside work,  Other reason for being Isolated,  Accepting the isolation,  Building alternative networks. | “You know I have never been invited to go play golf [...] I’ve never been invited on the ﬁshing trips, and I have never been invited to the ballgame.” -Female, Rabe-Hemp, 2007.  “When you’re a guy, you can rise through the ranks at a blistering pace based on no merit except on people you know, guys you play hockey with. And people say: ‘That’s a stand-up guy!’ . . . I don’t play hockey. I don’t even like hockey!” -Female, Murray, 2021.  “I have not experienced that in this department. Maybe it is because I got so used to it while I was in the military that I just don’t see it anymore.”-Female, Archbold & Schulz, 2008.  “We go out for breakfast or lunch and catch up on life and work stuff. It’s great to have so many women in leadership.” -Female, Murray, 2021. |
|  | **Navigating Acceptance from Team Members:**  Acceptance influenced by external influence,  Accepted on past performance,  Sexual preference plays role into acceptance,  Difficulty to earn acceptance in particular department,  Surpassing the discrimination during crisis period. | “I was at the top of my academy class for physical fitness. I could do more push-ups than all of the guys in my class. This got around the department before I even hit the streets. I think that I have earned their [male officers’] respect because of my level of physical fitness.”-Female, Archbold & Schulz, 2008.  “Lesbian females are more accepted by guys than straight females, because they are almost one of the guys. They can talk about the same stuff, they can joke about the same stuff, and the guys don’t feel like they are going to offend a lesbian female ... . they are very comfortable talking to me as a lesbian about other girls or stuff. . there is more of a comradery there.” -Female, Brown et al., 2019.  “We were really busy last year from a call out perspective. We had three shootings, three officer-involved shootings with my team. I had an officer that was shot last October. So, we hit the ground running and it didn’t take any time at all for these guys to feel like ‘yup, she’s got it. She knows what she is doing.’ So, internally I haven’t had any problems.” [SWAT Commander] -Female, Todak et al., 2022. |
|  | **Perceived Camaraderie:**  Gender-neutral feeling,  For long-term colleagues,  Dissatisfaction with the performance of the colleagues,  Backbiting the colleagues. | “I don’t think that gender is necessarily the reason that people feel camaraderie with other people. It has to do with how well the person can do their job.”- Female, Archbold & Schulz, 2008.  “could be male or female, completely whatever.”- Male, Murray, 2021.  “I see most of the ladies that have been here a while band together. We know what it is like being one of a few women so it is important to have others for support.” -Female, Archbold & Schulz, 2008.  “I complained of lack of cooperation among us as police members … no communication … working in this manner was not satisfactory”-Female, Gumani,2019. |
| Distributive Injustice | **Institutional Backlash Against Female Police Officers:**  Deprivation from organisational resources,  Treated as a 'Token Female Officer',  Crediting gender for career advancement,  Need to prove their worth,  Loss of morale & dedication,  Avoid asking for help in fear of getting judged,  Feeling of being sabotaged,  Negative impact on career. | “I was granted a position in Investigations for one year. They did not get me a desk, computer or even a phone with a contact number. I kept all of my stuff in a cardboard box for the entire year. Two weeks before I left Investigations there was a new desk, phone and computer set up for the guy coming in after I left.” - Female, Archbold & Schulz, 2008.  “A male’s idea or opinion could come across as more important that a female. So when a male speaks about something, they’re listened to. And a lot ofttimes when a female speaks [ . . . ] it’s overlooked or it’s like: “yeah, okay it’s a good idea but we’re not doing that”. -Female, Angehrn et al., 2021.  “When the local newspaper wanted to do an interview with someone from our department, the Chief told me that he wanted a female police officer to do the interview. I think that he wanted to showcase the fact that the department has a lot of women on the force. It wasn’t really about the diversification in the department; it was about his bragging rights.” -Female, Archbold & Schulz, 2008. |
|  | **Repercussions of Organisational & Operational Events on Health:**  Feeling unbefitting after return to work following injury,  Experience of dys-appearance leading to self-stigma,  Underreported mental illness,  Negative impact on mind, | “It’s just as if you’ve got brushed out, it’s seems as though they’ve done with you, you’ve done your little bit and oﬀ you go now because we can’t use you anymore. You’re supposed to be able to come to terms with that”. -Female, Bullock & Garland, 2019.  “As a result, mild bodily symptoms of stress at work, such as being ‘scrunched up’, older injuries ﬂaring up or twinges, aches and pains, were brushed off as unimportant, or became the subject of self-blame rather than institutional responsibility.” -Female,Yates et al., 2018  “This affected me a lot because it was my first case … a case of a 7-year-old … one of the cases that I worked on when I started working. I had not yet worked on other cases to have knowledge of other causes of the rape of young children, and why they were affecting me in that manner.” -Female, Gumani, 2019. |
|  | **Response to Evolving Life Situations:**  Compromising career advancement for family,  Difficulties due to caregiving responsibilities,  Restrictions on family planning,  Non-family-friendly working policies. | “So she [spouse of participant] basically put off her career to be able to get [child of participant] old enough that he could kinda look after himself sometimes. So I think that she made that sacriﬁce for sure and I think that would be pretty common for a lot of women in policing.”- Male, Angehrn et al., 2021.  “Well most women with five-to-seven years of service are having children so they go ‘there’s no way I could do that job, the overtime is ridiculous and you travel.’ So it doesn’t work for the most part. When you get up to my level and you look around the room, the women in the room mostly don’t have children, are lesbians with no children, are single for whatever reason, or they had their children very, very young before they joined.” -Female, Murray, 2021. |
|  | **Existing Support Initiatives & their Impacts:**  Variance in availability & utilisation of available support system,  Results of training on mental health,  Initiatives to support LGBTQ community. | “‘It felt like they were just completing a tick box and they weren’t interested at all. I think the initial support from them was poor really […] I don’t know why I bothered going to see them.”-Female, Bullock & Garland, 2019.  “Likewise, another officer commented he had seen a noticeable difference in the way line management’s attitudes were also changing. …the police are trying to do more, I think they are trying to recognise signs of stress in people, and what have you, that’s what they’re training line managers to do anyway, they are putting us on courses about stress management and things like that.”-Female, Edwards & Kotera, 2020. |
| Gendered Injustice | **External perceptions of female police officers:**  Bad ass woman on the front page,  Motivational figures,  Pressure to perform,  Gender perception by public,  Receiving negative attention,  Sexualisation of female officers. | “We would go to do our little manoeuvres in front of the parade. You could hear these gasps of mostly women saying, ‘it’s a girl!’” Another said “to citizens, it was more like seeing a white rhino at the zoo. Look honey, that’s a female motor officer.”-Female, Todak et al., 2022.  “I feel like I have to quickly take control of my calls so that the male officers know that I will respond quickly so that the situation doesn’t get out of hand. I don’t ever want them to assume that I wouldn’t step up and deal with my calls—all of them, even the ones that involve fights.”- Archbold & Schulz, 2008.  “The uniform takes away your race, it takes away your sex, and takes away everything about you. You are just a uniform, so they know you and know who you are. We are the enemy most of the time.” -Female, Rabe-Hemp, 2009.  “I always get hit on by citizens while I am on duty. I have asked some of the male cops if that ever happens to them and they have all told me that it doesn’t happen to them. I think that guys hit on me because I am wearing the uniform. I have also had a male citizen walk up to a table of me and four other female officers having lunch and say, “Is this all of the female cops that your department has?” I felt like I was on display or something.” -Female, Archbold & Schulz, 2008. |
|  | **Experiences related to reproductive health events:**  Impact on quality of life and work experience,  Stigma towards reproductive health events,  Discussion on menopause considered hyperbolic,  Embarrassed to disclose in male-dominated spaces,  Perceived gendered ageism,  Taking detrimental decisions in fear of being stigmatised. | “Raise my hand and say this is what I need... There is going to be that stigma, I did not feel comfortable making that approach at work and to say I need twenty minutes off, on a shift, [while out on patrol] I felt there was going to be that resistance” -Female, Newton & Huppatz, 2020.  “There is more emphasis on the menopause at the moment, it seems an ‘in’ term . . . If this goes too far it will be detrimental to women. We have fought hard for equality, but people will start to think [older] women . . . are less able to do their work, and a group apart.”- Female, Atkinson et al., 2020.  “‘Don’t be like some of these female ofﬁcers and just get pregnant as soon as you go 10-8.’ Which means don’t get pregnant as soon as you ﬁrst get on the road [patrol] and have to be at the front desk for 9 months. I looked at my one girlfriend, and we were like “Is this guy for fucking real?” - Female, Brown et al., 2019. |
|  | **Stigma Towards Mental Illness:**  Developing self-stigma,  Increased health risk from toxic work environment.  Normalising mental illness symptoms as ‘Part of the job’,  Ostracisation,  Fear of being stigmatised,  Fear of breach of confidentiality. | “I was paranoid, part of my anxiety was paranoia, so I thought that everybody hated me, I still get that sometimes now. You can get really worked up about what other people think about you.”. –Female, Edwards and Kotera, 2020  “Even some of the people who have been off with mental health or addiction issues, when they come back, they are treated horribly. People don’t give them a chance; they get ostracized. If you come back to work after a mental health issue and no one wants to know you or work with you–how do you get healthy? Who is going to willingly report after seeing stuff like that?”-Female, Bikos, 2021  “Although the organisation had an ‘ofﬁcial line’ on mental illness, the reality was quite different and that once a diagnosis of mental illness ‘got out [your] card was marked” -Female, Turner & Jenkins, 2019. |
|  | **Normalising sexism:**  Making inappropriate jokes,  Normalised as a mean to develop camaraderie,  Accepted as embedded characteristics,  Sexism towards women leaders from male subordinates. | “When I ﬁrst got hired, I had one ofmy sergeants tell me [ . . .] he said: “Just so you know, I’m a sexist, like don’t be offended by it, but I’m a sexist””. Female, Angehrn et al., 2021.  “Do I want to hear about ball scratching? No, I probably don’t. Does it make me feel bothered and offended? No. They treat you like you’re part of the club, you’re part of the boys’ team, you’re an equal .. . that you at least have the type of relationship where if they say something that offends you, you say it back to them and not go say a peep to a superior when they had no intention of making you feel that way .. . I don’t want people to feel like they have to treat me differently, so I take it as a compliment.” - Female, Brown et al., 2019.  “If I go in and tell someone “You need to do this” if I say it, then they say “ok”, and they’ll do it. If the women did the exact same thing with the same tone, it would be a different reaction. With a woman who’s exactly in the same position with exactly the same level of experience, it’s more of an uphill climb than [for] men.” –Male, Murray, 2021. |

**For Ovid:** (Health OR wellbeing OR well-being OR well being OR quality of life OR physical health OR mental health or physical health or injury or menopause or menstruation or reproductive health) AND (female OR women OR gender) AND (police officer OR law enforcement officer OR law enforcement OR cops OR police) AND (Qualitative study OR qualitative method OR qualitative research OR interview OR interviewing).

**For Proquest:** (Health OR wellbeing OR well-being OR well being OR quality of life OR physical health OR mental health) AND (female OR women OR gender) AND (police officer OR law enforcement officer OR law enforcement OR cops OR police) AND (Qualitative study OR qualitative method OR qualitative research OR interview OR interviewing) AND stype.exact("Scholarly Journals") AND at.exact("Article") AND la.exact("English") AND (subt.exact(("qualitative research" OR "police" OR "questionnaires" OR "perceptions" OR "law enforcement") AND ("interviews" OR "decision making" OR "aggression" OR "stress" OR "post traumatic stress disorder" OR "suicides & suicide attempts"))

**Figure S1: search terms used.**

Here, S1 shows the search terms used for finding the studies included in the review.

**Figure S2: Percentage of studies discussing organisational injustice and its relationship with various health aspect.**

Here, the Figure S2 shows the percentage of studies that explored different types of organisational injustice in connection to different health aspects (physical, mental, workplace wellbeing and social) relative to the total number of studies (twenty-one) included in the review.
